# Predicting Low-Risk Prostate Cancer from Transperineal Saturation Biopsies

**DOI:** 10.1155/2016/7105678

**Published:** 2016-04-11

**Authors:** Pim J. van Leeuwen, Amila Siriwardana, Monique Roobol, Francis Ting, Daan Nieboer, James Thompson, Warick Delprado, Anne-Marie Haynes, Phillip Brenner, Phillip Stricker

**Affiliations:** ^1^St Vincent's Prostate Cancer Centre, St Vincent's Clinic, Sydney, NSW 2010, Australia; ^2^Australian Prostate Cancer Research Centre New South Wales, The Garvan Institute of Medical Research, The Kinghorn Cancer Centre, Sydney, NSW 2010, Australia; ^3^University of New South Wales, Sydney, NSW 2052, Australia; ^4^Department of Urology, Erasmus University Medical Center, 3015 CE Rotterdam, Netherlands; ^5^Department of Public Health, Erasmus University Medical Center, 3015 CE Rotterdam, Netherlands; ^6^University of Notre Dame, Sydney, NSW 2010, Australia

## Abstract

*Introduction*. To assess the performance of five previously described clinicopathological definitions of low-risk prostate cancer (PC).* Materials and Methods*. Men who underwent radical prostatectomy (RP) for clinical stage ≤T2, PSA <10 ng/mL, Gleason score <8 PC, diagnosed by transperineal template-guided saturation biopsy were included. The performance of five previously described criteria (i.e., criteria 1–5, criterion 1 stringent (Gleason score 6 + ≤5 mm total max core length PC + ≤3 mm max per core length PC) up to criterion 5 less stringent (Gleason score 6-7 with ≤5% Gleason grade 4) was analysed to assess ability of each to predict insignificant disease in RP specimens (defined as Gleason score ≤6 and total tumour volume <2.5 mL, or Gleason score 7 with ≤5% grade 4 and total tumour volume <0.7 mL).* Results*. 994 men who underwent RP were included. Criterion 4 (Gleason score 6) performed best with area under the curve of receiver operating characteristics 0.792. At decision curve analysis, criterion 4 was deemed clinically the best performing transperineal saturation biopsy-based definition for low-risk PC.* Conclusions*. Gleason score 6 disease demonstrated a superior trade-off between sensitivity and specificity for clarifying low-risk PC that can guide treatment and be used as reference test in diagnostic studies.

## 1. Introduction

Early detection of prostate cancer (PC) has reduced PC mortality, but at the cost of a substantial increase in overdetection of insignificant disease [1, 2]. As a result, there are concerns about overdiagnosis and overtreatment of PC, and this has led to alternative diagnostic strategies and more conservative approaches, especially in the treatment of low-risk disease. Furthermore, as PC is most frequently a relatively slow-growing tumour, complications of unnecessary curative management in low-risk PC may be obviated by active surveillance (AS) [3]. Over the last decade, this has resulted in a growing acceptance of active surveillance for men diagnosed with low-risk PC [3].

Additionally, the emergence of multiparametric MRI (mpMRI) now allows imaging-based identification of PC, which improves diagnostic accuracy for higher-risk tumours, and might simultaneously reduce the diagnosis of low-risk PCs (overdiagnosis) [4, 5]. Considering the unreliability of PSA testing and 12-core template biopsy in PC diagnosis and risk stratification, the ability to visualise the tumour and then assess tumour grade and volume is of significant clinical benefit. However, to analyse the accuracy of mpMRI, a prospectively validated definition of clinically low-risk PC on saturation template mapping biopsy is required. mpMRI detects both high-grade and larger tumours accurately, which means it may perform particularly well for detection of clinically significant disease [4]. For diagnostic evaluation of mpMRI the ideal reference test would be definitive pathology of whole-mount sections of radical prostatectomy specimens. However, since such a reference test is not applicable in clinical practice, a reference test based on transperineal saturation biopsies would be useful [4].

There are multiple perspectives on low-risk PC (e.g., clinical, epidemiological, and pathological), and several definitions and terminologies are available [6]. The definition of “insignificant” or so-called “low-risk” PC, as well as the selection of men for AS, has been traditionally guided by clinicopathological features found to be predictive of indolent PC based on whole gland histopathology of radical prostatectomy (RP) specimens and defined as an organ-confined, well differentiated tumour (Gleason score ≤3 + 3 or ≤3 + 4) with a total tumour volume not exceeding 0.5–2.5 mL [7, 8]. Obviously, the use of the definition of pathologically insignificant PC requires an examination of the entire prostate. The translation, in clinical terms, of this histopathological definition of insignificant PC has proven to be challenging, owing to inherent inaccuracies of biopsy diagnoses. As a result, these definitions have been criticized for high rates of Gleason upgrading and unfavourable disease at RP [9–11]. Furthermore, it is unclear how to apply eligibility criteria based on 6–12 cores compared to men undergoing saturation biopsy [12–15].

The objective of this study was to assess the utility of five transperineal saturation biopsy-based clinicopathological risk criteria to predict insignificant PC in RP specimens. Thus the most accurate definition of low-risk PC based on transperineal saturation biopsies may be used as the endpoint (reference test) for future diagnostic imaging and biomarker studies. It may also be used to select candidate for AS after a PC diagnosis based on transperineal saturation biopsies.

## 2. Materials and Methods

### 2.1. Characterization of the Study Population

Since 2000, data from patients who underwent radical prostatectomy at our centre was collected prospectively in an institutional database. Institutional review board approval was granted and informed consent was obtained in all patients. For the present study, all men aged over 40 years, with PSA ≤10 ng/mL, diagnosed with a clinical T1c or T2, with Gleason <8 PC by transperineal template-guided mapping biopsy, between May 2001 and March 2015, and treated with a radical prostatectomy within 6 months of diagnosis, were included. All patients were reviewed and treated by two urologists at St Vincent's Clinic, Sydney, Australia.

### 2.2. Study Protocol

All men underwent biopsy for abnormal PSA or DRE. Transperineal template-guided mapping biopsy was performed with a median of 30 cores using 5 mm sampling of the peripheral zone and limited sampling of the transition zone with relative periurethral zone sparing adjusted for volume from 18 template locations. Histopathology from biopsies was processed and reported according to ISUP (International Society of Urological Pathology) protocols by one uropathologist. For all biopsies, the length of cancer was in millimetres (mm) and a primary and secondary Gleason grade were assigned to each cancer-containing core.

Subsequently all men underwent nerve sparing radical prostatectomy (open or robotic). Histopathology from radical prostatectomy specimens was processed following previously described protocols [16]. All specimens were fixed, inked, and cut at 3 mm intervals perpendicular to the rectal surface. The apical slice was cut parasagittally at 3 mm intervals. Pathological tumour stage, Gleason score, and surgical margin status were assessed. Tumour areas were marked for each slide and measured using 3D volume estimation.

### 2.3. Criteria Tested

A literature search was performed to identify published definitions for “insignificant”, “indolent”, or “low-risk” PC based on transperineal saturation biopsies. In total five clinicopathological definitions of low-risk PC were included and analysed, Table 1 [5, 8, 17]. In the present study, significant PC on radical prostatectomy specimen was defined, using the updated criteria, as PC with Gleason score 3 + 3 = 6 and a total tumour volume of ≥2.5 mL and as any PC of Gleason score 7–10 with greater than 5% Gleason grade 4 or higher and a total tumour volume of ≥0.7 mL [7].

### 2.4. Development Prediction Model

For descriptive statistical analysis, firstly the rates of significant PC with specific pathologically unfavourable PC characteristics, defined as the presence of either extracapsular extension, seminal vesicle invasion, lymph node invasion, or Gleason score 7–10 that fulfilled the proposed five criteria for low-risk disease, were assessed. Secondly, binary logistic regression was performed to explore the relationship between serum PSA, clinical stage, prostate volume, percentage of positive biopsy cores, and insignificant PC (yes/no) among all patients and individually for the patients that fulfilled the five criteria for low-risk disease. Sensitivity, specificity, negative predictive value (NPV), and positive predictive value (PPV) were assessed for all five criteria to predict insignificant PC in radical prostatectomy specimens.

The discrimination of the various criteria was tested using area under the curve of receiver operating characteristic (AUC-ROC) analysis. Discrimination describes the model's ability to differentiate between those with and those without the outcome of interest, in this case the presence of insignificant PC. *P* values were calculated to indicate whether the AUC was significantly different to the null hypothesis (AUC of 0.5).

Decision curve analysis (DCA) was used to plot the net clinical benefit, measured by weighing the benefits (true positive results) against the harms (false positives), at different probability thresholds, thus yielding the decision curve [18]. Decision curve analysis generated a graph of the net benefit as a function of a threshold probability (*p*_*t*_) at which an individual considers the potential benefit and harm of surgery to be equivalent. The threshold probability of insignificant cancer is an assumption where the patient would opt for AS. The decision curve is then compared with the two extremes of assuming all or no patients being treated with RP. The net benefit was measured as the rate at which incorporating the decision guide of interest (identifying low-risk PC) would lead to a beneficial decision to diagnose and treat PC without causing any additional harmful decisions to overtreat PC.

For all analyses, two-sided *P* values <0.05 were considered to be statistically significant. Statistical analyses were performed using SPSS version 21 and the R statistics package.

## 3. Results

Patient characteristics are presented in Table 2. Between May 2001 and March 2015, in total 3521 men underwent RP. 994 (28.2%) men fulfilled the inclusion criteria (PSA ≤ 10 ng/mL, diagnosed with clinical T1c or T2, Gleason <8 PC based on transperineal template-guided mapping biopsy) and were included for analysis. Of these men, 48 (4.8%) had a previous negative prostate biopsy. In 339 (34.1%) men a bilateral extended pelvic lymph node dissection was performed simultaneously to the RP. Of the 994 men who underwent RP, 143 (14.4%) fulfilled the criteria for insignificant PC at RP. Based on biopsy results, 52 (5.2%) men fulfilled criterion 1, 78 (7.8%) men fulfilled criterion 2, 188 (18.9%) men fulfilled criterion 3, 219 (22.0%) fulfilled men criterion 4, and 319 (32.1%) fulfilled criterion 5.

Table 3 shows the percentages of unfavourable pathological characteristics among the patients that fulfilled the five low-risk criteria. The number of men with a Gleason ≥4 + 3 at RP specimen and/or extracapsular disease was highest upon applying the least stringent criteria (criterion 5, 17.9%).

### 3.1. Risk Stratification

Multivariable logistic regression of preoperative clinicopathological parameters predicting insignificant PC at RP was performed. Significant variables predicting insignificant disease among all men included were lower PSA (OR 0.77, 95% CI 0.70–0.86), younger age (OR 0.92, 95% CI 0.92–0.98), larger prostate volume (OR 1.02, 95% CI 1.01–1.04), smaller percentage positive biopsies (OR 0.96, 95% CI 0.95–0.98), and a normal rectal examination (OR 0.56, 95% CI 0.37–0.85). The −2 log likelihood of the model was 689.35, specificity was 98.8%, sensitivity was 8.4%, and overall success rate was 85.8%. Regression coefficients (*B*) were −0.579 for normal rectal examination, −0.255 per ng/mL serum PSA, −0.080 per year of age, −0.039 per % positive biopsies, and 0.024 per cm^3^ prostate volume.

### 3.2. Performance of the 5 Transperineal Saturation Biopsy-Based Criteria

The ability of the five criteria to predict insignificant PC in RP specimen was examined, showing a sensitivity that ranged between 23% (criterion 1) and 82% (criterion 5) and a specificity that varied between 76% (criterion 5) and 98% (criterion 1). The sensitivity, specificity, PPV, NPV, and LR for all five criteria are presented in Table 4. Receiver operator characteristics to assess the performance of the five biopsy-based criteria, to predict insignificant PC in RP, are shown in Table 5. The AUC for each model ranged between 0.604 (criterion 1), 0.654 (criterion 2), 0.741 (criterion 3), 0.790 (criterion 5), and 0.792 (criterion 4), suggesting moderate positive discrimination at best.

A DCA was performed to assess the clinical utility of using each of the five criteria for the definition of low-risk PC to guide the decision of who should not be treated (in order to decrease overtreatment) and/or be selected for AS. Two additional criteria (treatment with RP to all and treatment to none) were added for comparison. The DCA showed that, for threshold probabilities of 0% to 15%, representing a preference to maximize reduction in overtreatment, the net benefit is greatest using criterion 5. In this range of threshold probabilities, patients appear to be more concerned about overtreatment than about missing significant PC. For the midrange, as well as clinically most relevant probabilities of 30% to 50%, criterion 4 was superior to the other criteria. For the higher thresholds (50–75%), at which patients may be more concerned about missing a significant cancer than about unnecessary overtreatment, the more rigorous criteria 1 and 2 should be used. Using a threshold of >75% is best to diagnose and/or treat all patients.

## 4. Discussion

“Overdiagnosis” and “overtreatment” are defined as the diagnosis and/or treatment of PC that would never cause symptoms or death during the patient's lifetime and may therefore be considered unnecessary [19]. Obviously, both definitions are influenced by disease-related factors and by the natural life expectancy of a patient. “Indolent” PC is defined as a cancer that never becomes locally invasive or metastatic leading to symptoms or death, irrespective of the patient's comorbidities or expected lifespan [6, 8]. This is in contrast to the term “insignificant,” which is epidemiologically defined and based on lifetime risk estimations of the occurrence of symptomatic or clinical PC in a population. Consequently, a tumour classified as nonindolent in a patient who dies of an unrelated cause and has not experienced morbidity from PC will also be classified as overdiagnosed. Overdiagnosis has been estimated between 22 and 67% using different screening regimens [2]. In this context, defining low-risk or insignificant PC in a considerable percentage of PCs is an important diagnostic step. In our study cohort, using five different biopsy-based criteria, 6% to 32% of men were classified as having low-risk PC.

**Figure 1:**
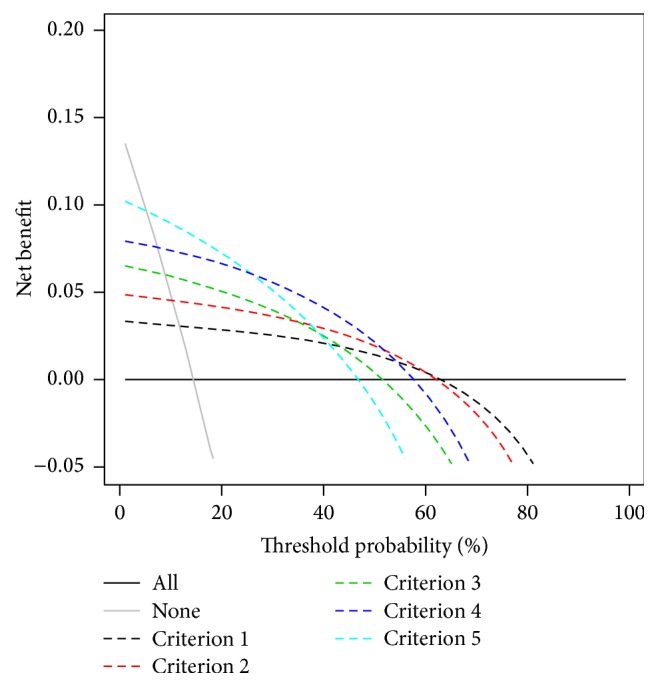
Decision curve analysis, demonstrating the net benefit, as measured by rate of nontreating men for low-risk prostate cancer, using the five decision-making strategies as listed in the legend. Threshold probability is the threshold probability of low-risk PC at which an individual considers the benefit of treatment for PC equivalent to the harm of overtreatment for low-risk disease, and thus it reflects how the individual weights the benefits and harms associated with this decision. The highest curve at any given threshold probability is the optimal decision-making strategy to maximize net benefit.

In outlining a definition for low-risk PC to reduce overdiagnosis and minimize morbidity associated with overtreatment of PC, the ability to “accurately” select men is paramount. In the present study, we analysed five criteria to predict men with insignificant PC at RP specimen, a surrogate for low-risk PC and/or suitability for AS. Not surprisingly, the specificity was better among the more stringent criteria for low-risk PC (criteria 1 and 2). In contrast the AUCs were found to be significantly higher among the less stringent criteria (criteria 4 and 5). For our specific research question, specificity might take precedence over sensitivity and therefore a DCA was performed to determine clinical utility (Figure 1). Based on clinical acceptance, the criteria with the highest sensitivity by a specificity of 90% would be assumed as the optimal definition for clinical use [2, 3]. As a result, criteria 3 and 4 may be used for further patient selection and validation purposes.

Furthermore, it should be noted that the benefits of early detection and active treatment are limited, and ultimately what is required is improved selection of patients with significant PC who will benefit from immediate active treatment [1, 2]. In this study, it was observed that the application of more liberal criteria would enable 3.6-fold more men to participate in AS program, resulting in a 10% increased risk of underestimating a significant PC (criterion 3 versus criterion 1). In other words, among the present study population, we would decrease the “overdiagnosis” by 13.7% if we only diagnosed insignificant PC using criterion 3 (18.9%) versus criterion 1 (5.2%). In our opinion, these trade-offs could be beneficial, in terms of both quality of life and financial costs related to the early detection of PC [20]. Finally, given the slow evolution of PC, a deferment in definitive treatment would not be likely to substantially alter disease course or limit the patient's chance of complete cure [21].

Finally, the results also showed that, even with the most stringent selection criteria, it is impossible to perfectly differentiate between clinically insignificant and possible life-threatening PC, even after a diagnosis based on transperineal saturation biopsies. Suardi et al. reported rates of 24 to 27.8% of unfavourable PC characteristics in a cohort based on 2 of the most “stringent” criteria looking at 12-core TRUS guided biopsies [9]. Other series reported rates of 8.4–30.5% of unfavourable outcomes in a 12-core TRUS population fulfilling the Epstein criteria [11, 22, 23]. Ultimately, to accurately identify insignificant cancer we will need to look beyond histopathology in isolation, integrating biopsy data with the high-resolution 3D mapping and functional information derived from mpMRI and possibly that from biomarkers (PCA3, pro-2 PSA, and TMPRSS2:erg), mitochondrial RNA mapping (e.g., PC Mitomics Test), epigenetic DNA mapping (e.g., MDx Link), and other emerging predictors of the biologic behaviour of PC in an individualised way [24–28].

Results from the present study may be used for further development of mpMRI protocols. For evaluation of mpMRI performance, ideally inclusion of definitive pathology is required from whole-mount sections of RP specimens as the reference standard [4]. However, since such study designs are not applicable in clinical practice due to the selection biases created by the absence of RP specimens in patients who have no cancer on biopsy low-risk cancer managed with AS or high-risk cancer managed with radiotherapy, an alternative approach is to use transperineal saturation template prostate mapping biopsy instead [4]. Transperineal saturation template mapping biopsy outperforms TRUS guided prostate biopsies, showing an average upgrading in 33% of patients and a change from unilateral to bilateral disease in more than 50% of patients [29]. The present study aids the interpretation of the outcomes of this alternative and demonstrates that saturation biopsy is not a perfect reference standard. Biopsy criterion 3 and criterion 4 showed a specificity and NPV of 86–88% and 93–95%, respectively, for accurately predicting insignificant PC in RP, and therefore may represent clinically relevant definitions for selection of active surveillance patients.

The present study is subject to limitations. Firstly, this was a retrospective analysis of prospectively collected data from only two surgeons in a single centre. Secondly, there is a major selection bias towards higher-risk patients, given that the cohort consists entirely of patients who underwent RP, a decision which may have been driven by unmeasured clinical factors such as a rapidly rising PSA or significant lesions on mpMRI. This could negatively bias the accuracy estimates for each definition. It is a highly selected study population with no control arm. Furthermore, given the long period over which this study data was collected, changes in Gleason reporting for PC may also represent a confounder regarding biopsy and RP specimen pathology. Also, diagnostic performance in terms of sensitivity and specificity are subject to inclusion criteria. We included only low-risk men diagnosed with clinical stage ≤T2, PSA <10 ng/mL, and Gleason score <8 PC in order to estimate the performances of the diagnostic criteria in low-risk patients. Diagnostic performance may differ in higher-risk patients, who are less likely to harbour insignificant disease and are not usually candidates for AS. Furthermore, transperineal saturation biopsies are not generally accepted as a reference test [4], and it is still unclear if transperineal saturation biopsies might detect more insignificant cancers compared to a standard biopsy. Finally, prospective studies that use these biopsy definitions to select patients for AS and then evaluate long-term oncological outcomes will provide a higher level of evidence. However, until such studies are published, retrospective studies such as this can be used to guide patient counselling and treatment decisions.

In conclusion, balancing overtreatment of insignificant PC with undertreatment of significant PC is an ongoing challenge. This study analysed the performance characteristics of various criteria at predicting insignificant PC on transperineal template-guided saturation mapping biopsies in a relatively low-risk PC population who underwent RP. No single definition was superior; however, patients with Gleason 6 disease demonstrated the best trade-off between sensitivity and specificity for clarifying low-risk PC. Depending on patients and physicians preferences, these criteria can be used for treatment decisions and patient counselling and as a reference test in diagnostic studies.

## Figures and Tables

**Table 1:** Five transperineal saturation biopsy-based criteria for low-risk prostate cancer.

Criteria	Definition
Criterion 1	Gleason score 6 + ≤5 mm total max core length PC + ≤3 mm max per core length PC
Criterion 2	Gleason score 6 + <20% of cores positive + <5 mm max core length PC
Criterion 3	Gleason score 6-7 with ≤5% Gleason grade 4 + <20% of cores positive + <7 mm max core length PC
Criterion 4	Gleason score 6
Criterion 5	Gleason score 6-7 with ≤ 5% Gleason grade 4

**Table 2:** Patients characteristics.

Variable	All	Criterion 1	Criterion 2	Criterion 3	Criterion 4	Criterion 5
Number of patients	994	52	78	127	219	319
Median (IQR)						
Age	62	60 (57–63)	60 (56–64)	60 (55–64)	59 (55–63)	60 (55–64)
PSA ng/mL	5.5	5.5 (4.0–7.2)	5.5 (4.2–6.8)	5.5 (4.1–7.0)	5.1 (3.8–6.3)	5.1 (3.8–6.3)
Prostate volume	49.0	53.0 (45–69)	53.0 (44–70)	52.0 (44–62)	49.0 (41–57)	48.0 (40–56)
Number of cores	27	28	28	28	28	28
% positive cores	25.0	9.5	11.1	12.5	18.8	21.4
Clinical stage, *N* (%)						
T1c	537 (54.0)	43 (82.7)	62 (79.5)	93 (73.2)	151 (68.9)	208 (65.2)
T2a	312 (31.4)	5 (9.6)	11 (14.1)	22 (17.3)	47 (21.5)	73 (22.9)
T2b	72 (7.3)	1 (1.9)	2 (3.8)	3 (2.4)	11 (5.0)	15 (4.7)
T2c	73 (7.3)	3 (5.8)	3 (2.6)	9 (7.1)	10 (4.6)	23 (7.2)
Biopsy Gleason score, *N* (%)						
3 + 3	221 (22.2)	52(100)	78 (100)	99 (78.0)	219 (100)	218 (68.3)
3 + 4	581 (58.5)			28 (22.0)		101 (31.7)
4 + 3	192 (19.3)					

**Table 3:** Unfavourable pathological characteristics per biopsy criteria.

Variable	Criterion 1	Criterion 2	Criterion 3	Criterion 4	Criterion 5
Number of patients	52	78	127	219	319
Gleason score					
4 + 3 (%)	1 (1.9)	2 (2.6)	1 (0.8)	3 (1.4)	5 (1.6)
≥8 (%)	0 (0.0)	0 (0.0)	2 (1.6)	1 (0.3)	3 (0.9)
ECE (%)	4 (7.7)	6 (7.7)	11 (8.7)	28 (12.8)	55 (17.2)
SVI (%)	0 (0.0)	0 (0.0)	0 (0.0)	0 (0.0)	1 (0.3)
LNI (%)	0 (0.0)	0 (0.0)	0 (0.0)	0 (0.0)	0 (0.0)
Any unfavourable pathological characteristics	5 (9.6)	7 (8.9)	13 (10.2)	29 (13.2)	57 (17.9)

**Table 4:** The ability of the five criteria to predict insignificant PC in RP specimen.

	Specificity	Sensitivity	PPV	NPV
Criterion 1	98	23	63	88
Criterion 2	97	34	63	90
Criterion 3	91	52	46	93
Criterion 4	86	72	47	95
Criterion 5	76	82	37	96

**Table 5:** Outcomes of area under the receiver operating characteristic analysis of the five biopsy criteria.

	AUC	95% CI	*P *value
Criterion 1	0.604	0.549–0.660	<0.01
Criterion 2	0.654	0.599–0.712	<0.01
Criterion 3	0.641	0.641–0.749	<0.01
Criterion 4	0.792	0.747–0.837	<0.01
Criterion 5	0.790	0.750–0.831	<0.01
